# Letrozole Reduces Ovulatory Responsiveness in In Vitro‐Grown Mouse Follicles

**DOI:** 10.1002/rmb2.70047

**Published:** 2026-04-15

**Authors:** Tatsuya Kobayashi, Hiroshi Ishikawa, Hirokazu Tanaka, Kumiko Ishii, Akira Mitsuhashi, Hirokazu Usui, Makio Shozu, Kaori Koga

**Affiliations:** ^1^ Research Promotion Unit, School of Medical Sciences Fujita Health University Tokyo Japan; ^2^ Department of Obstetrics and Gynecology, Reproductive Medicine, Graduate School of Medicine Chiba University Chiba Japan; ^3^ Saisei Hospital Chiba Japan; ^4^ Department of Obstetrics and Gynecology, School of Medicine Dokkyo Medical University Tochigi Japan

**Keywords:** breast cancer; letrozole, luteinizing hormone/chorionic gonadotropin receptor, ovulation

## Abstract

**Purpose:**

This study aimed to determine whether continuous aromatase inhibition with letrozole reduces peri‐ovulatory responsiveness to ovulatory stimulation in in vitro‐grown mouse follicles.

**Methods:**

Early antral follicles were isolated from 21 to 24‐day‐old C57BL/6jjcl mice and cultured individually for 5 days with letrozole (0.01, 0.1, or 1 μM) or vehicle. Follicle survival and growth were assessed by measuring follicle diameter. Ovulatory responsiveness was evaluated by hCG/EGF stimulation. Expression of ovulation‐related genes (*Lhcgr*, *Ptgs2*, and *Runx1*) and a luteinization‐related gene (*Foxo1*) was analyzed using RT‐qPCR. The effect of estradiol supplementation was also examined.

**Results:**

Letrozole did not affect follicle survival or growth. However, ovulation rates were reduced in a dose‐dependent manner (*p* < 0.0001). At 0.1 μM, letrozole significantly decreased mRNA levels of *Lhcgr*, *Runx1*, and *Ptgs2* compared with vehicle‐treated follicles. Estradiol supplementation restored the expression of these genes and partially rescued ovulatory capacity. In contrast, *Foxo1* expression increased in letrozole‐treated follicles and was attenuated by estradiol.

**Conclusions:**

Continuous letrozole exposure reduces ovulatory responsiveness in in vitro‐grown mouse follicles and is associated with decreased Lhcgr expression after hCG stimulation, likely due to estrogen deficiency. These findings suggest that prolonged aromatase inhibition during follicular growth may impair acquisition of peri‐ovulatory competence.

## 1 Introduction

Fertility preservation for young cancer survivors has become commonplace worldwide due to the development of assisted reproductive technologies such as oocyte cryopreservation, embryo cryopreservation, and ovarian tissue freezing. Breast cancer is the most common cancer among women, with approximately 2%–10% of cases occurring annually in women of reproductive age [1, 2]. Recent advances in anticancer therapy for breast cancer improve the 10‐year survival rates of breast cancer patients in adolescent and young adult age by up to 70%, leading to many survivors who wish for a baby in the future [1, 2]. Since many anticancer agents for breast cancer, such as cyclophosphamide, doxorubicin, and epirubicin hydrochloride, have ovarian toxicity, some women experience iatrogenic menopause after treatment. Therefore, fertility preservation using assisted reproductive technologies helps these patients retain the possibility of having children using their oocytes [3].

Aromatase, encoded by *CYP19A1*, is a single enzyme that catalyzes the conversion of androgen to estrogen [4]. Only mural granulosa cells express aromatase at the preovulatory stage in the ovary, and this expression is regulated by gonadotropins from the pituitary gland and several local ovarian factors [5]. Letrozole, a third‐generation aromatase inhibitor, is a drug that has been approved to be an anticancer agent for postmenopausal women with breast cancer as well as for ovulation induction in women with polycystic ovary syndrome and unexplained infertility [6]. It is also used in controlled ovarian stimulation (COS) cycles in fertility preservation programs to keep serum estrogen levels low in women with breast cancer. It is administered daily at the same time as gonadotropin injection or from the day after gonadotropin injection until the day before ovulation triggering. The 5‐year survival rate of patients undergoing fertility preservation using COS with letrozole did not differ from that of patients without fertility preservation [7]. Therefore, the letrozole combination COS has been considered a safe fertility preservation program in women with estrogen receptor (ER)‐positive breast cancer, in terms of the absence of an increased recurrence risk of cancer currently [7, 8, 9, 10]. However, early clinical observations indicated that when conventional trigger criteria were applied during continuous letrozole coadministration, an increased proportion of immature oocytes was encountered, and this improved after adjusting the timing of hCG administration [7]. In addition, Sonigo et al. reported a reduced number of cryopreserved mature oocytes in cycles with continuous letrozole supplementation [11]. These observations suggest that continuous aromatase inhibition may influence the acquisition of peri‐ovulatory competence, rather than simply affecting follicular growth.

Estrogen acts as a paracrine factor that promotes the proliferation of endometrial cells and regulates gonadotropin release in female reproductive cycles. It also acts as an autocrine factor in follicle development. The effects of aromatase and estrogen on folliculogenesis and oocyte maturation have been well studied in female aromatase knock‐out (ArKO) mice, which lack endogenous estrogen. Female ArKO mice are infertile due to an ovulation disorder [12]; however, ArKO oocytes have normal maturation ability in vitro, and in vitro‐matured oocytes have normal preimplantation development after fertilization [13, 14]. ArKO mice also showed decreased luteinizing hormone/chorionic gonadotropin receptor (*Lhcgr*) transcription with reduced activities of its downstream signaling pathways, such as extracellular signal‐regulated kinase 1/2, in the ovary after ovulation triggering [15]. Female estrogen receptor β knock‐out (ErβKO) mice, which have a similar ovulation disorder as ArKO mice, represent an estrogen‐deficient model to study estrogen action on ovulation and oocyte maturation [16]. Rodriguez et al. reported that ErβKO mice have low *Lhcgr* transcription in follicles and Erβ‐mediated signaling is required for *Lhcgr* expression in mural granulosa cells that receive a luteinizing hormone (LH) surge for ovulation [17]. The final Lh‐Lhcgr signaling induced by LH surge is necessary for ovulation as well as cumulus cell expansion and oocyte maturation. These findings suggest that letrozole may alter peri‐ovulatory follicular responses in mural granulosa cells.

In this study, we used an in vitro cultured mouse follicle model to examine the effects of continuous letrozole exposure throughout follicular growth on follicle development and subsequent responsiveness to ovulatory stimulation. We aimed to determine whether sustained aromatase inhibition during this developmental window alters follicle growth and peri‐ovulatory follicular responses, in light of findings from ArKO and ErβKO mice.

## 2 Materials and Methods

### 2.1 Animals and Follicle Isolation

**FIGURE 1:**
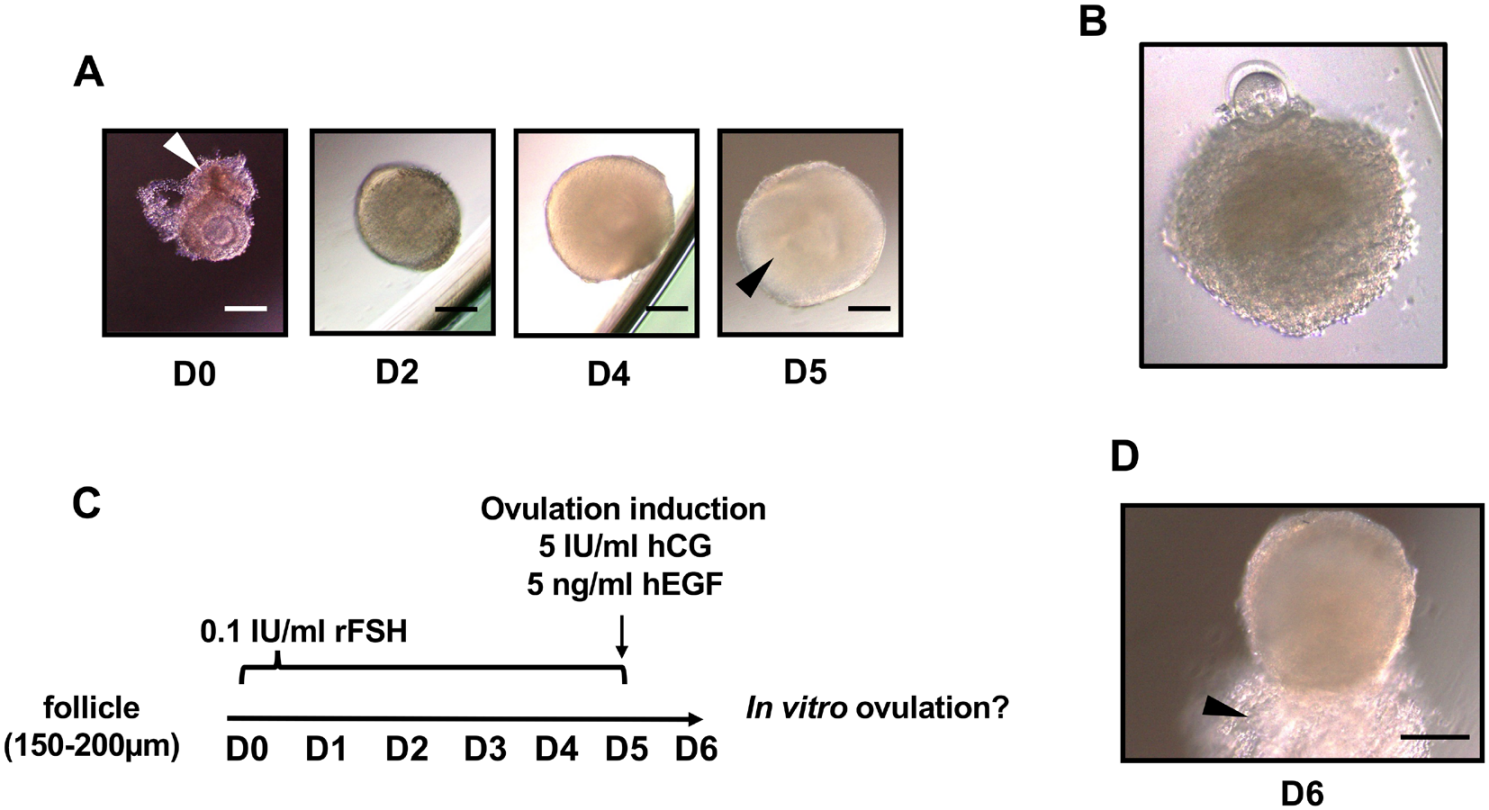
In vitro growth and ovulation of mouse early antral follicles. (A) Follicles collected from immature mouse ovaries were cultured for 5 days in vitro. There were multilayer granulosa cells and a small number of stromal cells (white arrow). Antral cavity (black arrow) formed within the granulosa cell layer. (B) A follicle with an abnormal basement membrane was judged as an atretic follicle. (C) Time course of the in vitro culture in this study. (D) In vitro ovulated follicle. The expanded cumulus–oocyte complex was released from the follicles (black arrow). hCG, human chorionic gonadotropin; rFSH, recombinant follicle‐stimulating hormone; hEGF, human epidermal growth factor.

C57BL/6jjcl female mice were obtained from CLEA Japan Inc. (Tokyo, Japan). The mice were maintained in a temperature‐ and light‐controlled room. We isolated the ovaries from two 21‐to‐24‐day‐old female mice and placed them in Leivobitz's‐L15 medium (Thermo Fisher Scientific, Waltham, USA), supplemented with 3 mg/mL bovine serum albumin (Sigma‐Aldrich), 5 μg/mL bovine insulin (Sigma‐Aldrich, St Louis, Missouri), 10 μg/mL human apo‐transferrin (Sigma‐Aldrich), 2 ng/mL sodium‐selenite, 5 ng/mL ascorbic acid (Sigma‐Aldrich), and 1% gentamicin (Invitrogen, Waltham, Massachusetts). Follicles with diameters of 150–200 μm and a few stromal cells (Figure 1A) were mechanically isolated from the ovary using a 31‐gauge needle under a stereo microscope and collected in a culture medium. The isolated follicles correspond to late secondary to early antral follicles in mice, based on established morphological classifications of murine folliculogenesis [18]. These stages may be functionally comparable to small antral follicles observed during the early follicular phase in women [19], as both are characterized by the acquisition of follicle stimulating hormone (FSH) responsiveness. Thus, our model conceptually reflects the period in which small antral follicles acquire peri‐ovulatory competence under FSH exposure prior to an ovulatory trigger, although direct temporal and size equivalence between species cannot be assumed.

### 2.2 In Vitro Culture of Mouse Early Antral Follicles

For follicle culture, we used phenol red‐free α‐MEM Gluta‐MAX (Thermo Fisher Scientific) supplemented with 0.1 IU/mL recombinant human FSH (rFSH, follistim, MSD, Tokyo, Japan), 5 μg/mL bovine insulin (Sigma‐Aldrich, St Louis, Missouri), 5 μg/mL human apo‐transferrin, 5 ng/mL sodium‐selenite, 5 ng/mL ascorbic acid (Sigma‐Aldrich), 2 mM sodium pyruvate (Wako, Osaka, Japan), and 5% charcoal dextran‐treated fetal bovine serum. Follicles were individually placed in ultra‐low attachment 96‐well plates (Corning, New York, NY, USA), filled with 200 μL/well of culture medium and without mineral oil. Since letrozole (Sigma‐Aldrich) is insoluble in water, it was dissolved in dimethyl sulfoxide (DMSO; Sigma‐Aldrich) and then added to the culture medium. All the experiments, including the untreated control (vehicle), were performed in 0.1% DMSO. Follicles were cultured for 5 days at 37°C under 5% CO_2_ and 100% humidity (Figure 1A). Medium refreshment was performed every 2 days by replacing 100 μL of old medium with fresh medium. Follicles with an abnormal basement membrane on Days 2 and 5 were considered nonsurvival follicles (Figure 1B). The survival rate of each group was calculated by number of survival follicles/total number of cultured follicleles (%). The follicular size was measured using ImageJ (U.S. National Institutes of Health, Bethesda, MD, USA). Immediately after isolation, follicle diameter was determined as the mean of two perpendicular measurements taken between basement membranes. From Day 2 onward, diameter was determined as the mean of two perpendicular measurements of the outer follicle outline. We also calculated the proportion of “mature follicles” (≥ 300 μm and morphologically normal) among all cultured follicles in each group.

### 2.3 Steroid Hormone Assay

To measure steroid hormone levels in cultured media, cultured media derived from three follicles on Day 5 were pooled and estradiol concentrations were measured in Day 5 cultured media using an Estradiol ELISA Kit (Cayman CHEMICAL, Ann Arbor, MI, USA) and Infinite‐m200 (Tecan Japan, Kanagawa, Japan) according to the manufacturer's protocol.

### 2.4 In Vitro Ovulation Assay

On Day 5 of the follicle culture, all media were replaced with fresh medium containing 5 IU/mL human chorionic gonadotropin (hCG; Asuka Animal Health, Tokyo, Japan) and 5 ng/mL recombinant human epidermal growth factor (hEGF; Sigma‐Aldrich) to induce in vitro ovulation. Ovulation was evaluated in the follicles that ruptured and released a cumulus–oocyte complex (COC) into the medium after 16 h of hCG and hEGF stimulation (Figure 1D). Ovulation rate was defined as the number of ovulated follicles divided by the total number of morphologically normal follicles with a diameter ≥ 300 μm at the time of hCG stimulation.

### 2.5 Total RNA Extraction and Real‐Time Quantitative Polymerase Chain Reaction (RT‐qPCR)

Total RNA from cultured follicles was extracted using the RNeasy Micro Kit (Qiagen, Hilden, Germany) according to the manufacturer's protocol. For time‐course analysis of *Cyp19a1* and *Lhcgr* expression in the vehicle group, follicles were collected on culture Days 0, 2, 4, and 5.

For analysis of peri‐ovulatory gene expression (e.g., *Lhcgr*, *Ptgs2*, *Runx1*, *Foxo1*), follicles were collected 16 h after hCG/EGF stimulation. For each biological replicate, 2–3 follicles were pooled and lysed in 350 μL of Buffer RLT (Qiagen), and this pooled sample was defined as *n* = 1. The extracted total RNA was reverse‐transcribed into complementary DNA using the SuperScript VILO cDNA synthesis kit (Thermo Fisher Scientific). RT‐qPCR was performed using a LightCycler DNA Master SYBR Green I Kit (Roche, Basel, Switzerland) on a Light Cycler 2000 system. We evaluated *Cyp19a1*, *Lhcgr*, and *Fshr* expression during the culture period. To evaluate peri‐ovulatory gene expression after hCG/hEGF stimulation in cultured follicles, we evaluated *Lhcgr*, prostaglandin‐endoperoxide synthase 2 (*Ptgs2*), and Runt‐related transcription factor 1 (*Runx1*) as ovulation‐related genes. We also evaluated Foxo1 as a luteinization‐related gene. β‐actin (*Actb*) transcription was used as an internal control. The −∆*C*_t_ value for each target gene was calculated as ∆*C*_t_ (*Actb*) − ∆*C*_t_ (target gene). The −∆*C*_t_ values of the experimental groups were compared with those of the vehicle‐treated group (vehicle group). A higher −∆*C*_t_ value indicates higher mRNA expression. The gene‐specific primers are shown in Supplemental Table S1.

### 2.6 Statistical Analysis

At least two independent experiments were performed. All results were analyzed by pooling the results of independent experiments. Statistical analysis was performed using JMP Pro 15 statistical software (SAS Institute Inc., Cary, NC, USA). The survival rates and ovulation rates at different letrozole concentrations were compared using the Cochran–Armitage test. RT‐qPCR results were analyzed using the Wilcoxon test. *p* < 0.05 was considered statistically significant. The correlation between the −∆*C*_t_ values of each gene was evaluated using Pearson's correlation coefficient.

## 3 Results

### 3.1 The Transcription of *Cyp19a1* and *Lhcgr* in In Vitro Follicle Growth

**FIGURE 2:**
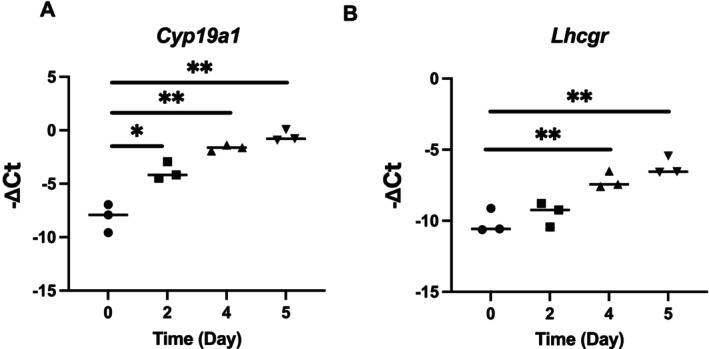
Expression levels of *Cyp19a1* and *Lhcgr* transcripts in in vitro‐cultured mouse follicles. The mRNA expression levels of *Cyp19a1* (A) and *Lhcgr* (B) in the whole follicles were measured using real‐time quantitative polymerase chain reaction (RT‐qPCR) at Days 0, 2, 4, and 5 of the culture period (*n* = 3 per group). −∆*C*_t_s of *Cyp19a1* and *Lhcgr* were calculated using the Ct value of *Actb*. *p < 0.05, **p < 0.01, comparisons with the vehicle (letrozole‐free) group. Actb, β‐actin; hCG, human chorionic gonadotropin; Lhcgr, luteinizing hormone/chorionic gonadotropin receptor; rFSH, recombinant follicle‐stimulating hormone.

The −∆*C*_t_s of *Cyp19a1* and *Lhcgr* during the culture period are shown in Figure 2A,B. The −∆*C*_t_ of *Cyp19a1* increased during the culture period. In contrast, the −∆*C*_t_ of *Lhcgr* increased significantly on Days 4 and 5 as compared with that on Day 0 of culture.

### 3.2 Effect of Letrozole on Follicle Survival and Changes in Follicle Diameters In Vitro

**FIGURE 3:**
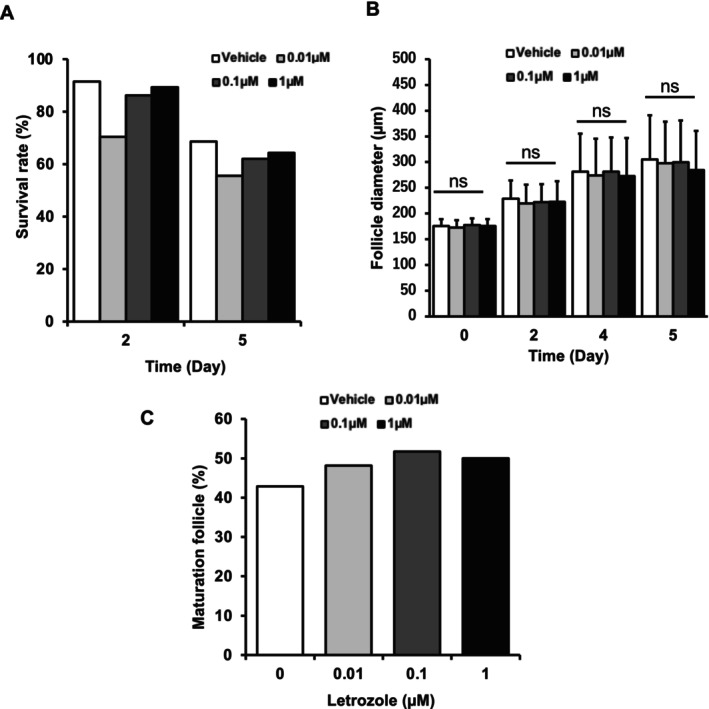
Effect of letrozole on the survival and growth of in vitro‐cultured mouse follicles. (A) Letrozole did not affect follicular viability (*n* = 35, 27, 29, 28). (B) Early antral follicles (150–200 μm) collected from 3‐week‐old C57BL/6jjcl mice were cultured in the presence of letrozole (0.01–1 μM), and the follicle diameter was measured. At this time, the letrozole‐free group was used as a control (*n* = 35, 27, 29, 28). The follicle diameters were presented as mean ± standard deviation. (C) Mature follicles were defined as morphologically normal follicles with a diameter ≥ 300 μm at the time of stimulation. The percentage was calculated as (number of mature follicles/total number of cultured follicles). The number of cultured follicles were 35, 27, 29, and 28 in the 0, 0.01, 0.1, and 1 μM letrozole groups, respectively.

The survival rates of letrozole‐treated follicles were not significantly different as compared with those of vehicle‐treated follicles (Figure 3A). Letrozole did not affect follicular viability when trend tests were performed. When mouse follicles were cultured using our follicle culture protocol without letrozole for 5 days, they grew to approximately 300 μm from approximately 170 μm (Figure 3B). Similarly, as shown in Figure 3B, follicles cultured with several concentrations of letrozole (0.01 μM, 0.1 μM, or 1.0 μM) also grew to the same size as vehicle‐treated follicles. The proportion of mature follicles was 42.9% (15/35) in the vehicle group, 48.2% (13/27) in the 0.01 μM letrozole treated group, 51.7% (15/29) in the 0.1 μM letrozole treated group, and 50.0% (14/28) in the 1 μM letrozole treated group (Figure 3C). Although the proportion numerically increased up to 0.1 μM, the Cochran–Armitage trend test did not detect a significant dose‐dependent trend.

### 3.3 Effect of Letrozole on Estrogen Production

Estradiol concentrations in the culture medium were measured on Day 5. In the vehicle group, estradiol levels were 175.2 ± 60.0 pg/mL (mean ± SD; *n* = 3 independent experiments). In the 0.1 μM letrozole‐treated group, E2 concentrations were below the assay limit of detection (20 pg/mL).

### 3.4 Effect of Letrozole on Ovulatory Responsiveness In Vitro

**FIGURE 4:**
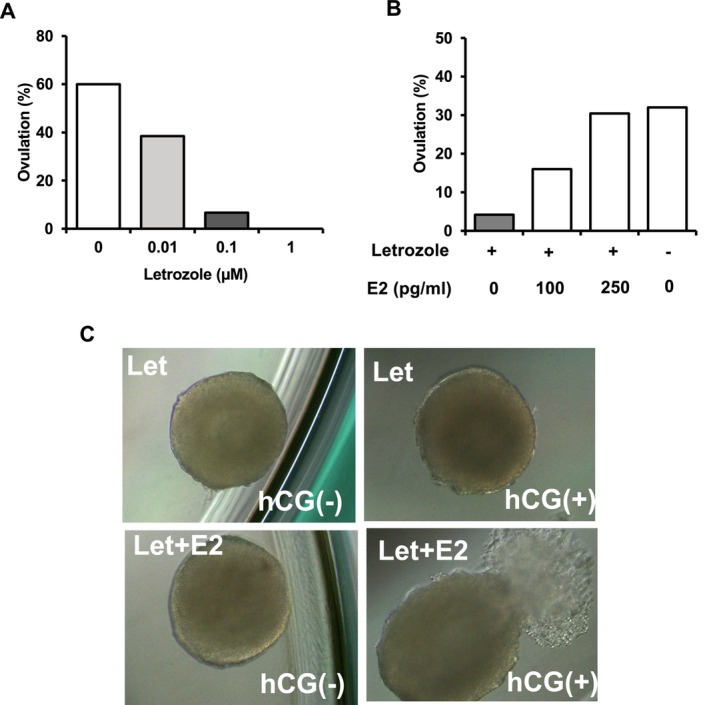
Effects of letrozole on in vitro ovulation. (A) The cultured morphologically normal follicles with a diameter > 300 μm were stimulated using 5 IU/mL hCG and 5 ng/mL hEGF on day 5 to evaluate ovulatory responsiveness. In vitro ovulation was assessed after 16 h after ovulation induction. The addition of letrozole reduced ovulation in a dose‐dependent manner (*n* = 15, 13, 15, 14, Cochran–Armitage trend test, *p* < 0.0001). (B) Effect of exogenous estrogen on letrozole‐treated follicles. Follicles previously treated with 0.1 μM letrozole were stimulated with 100 pg/mL or 250 pg/mL estradiol from Day 4 to Day 5 of the culture period. Exogenous estrogen improved the ovulation rate of 0.1 μM letrozole‐treated follicles from 4.2% to 16% and 30% (*n* = 24, 27, 23, 25, Cochran–Armitage test, *p* < 0.01). (C) The upper two panels show letrozole‐treated follicles before and after hCG stimulation. The lower two panels show letrozole and estradiol‐treated follicles before and after hCG stimulation. Estrogen treatment restored in vitro ovulation of letrozole‐treated follicles. E2, estradiol; hCG, human chorionic gonadotropin; hEGF, human epidermal growth factor.

To evaluate the ovulatory responsiveness of letrozole‐treated follicles, we analyzed whether in vitro ovulation could be induced by hCG and hEGF treatment as an ovulation trigger in the culture. We stimulated cultured morphologically normal follicles with diameters > 300 μm with 5 IU/mL hCG and 5 ng/mL hEGF for 16 h on Day 5 of the culture. In the vehicle group, 60.0% of follicles released COC into the medium after ovulation induction (9/15, Figure 4A). On the other hand, letrozole reduced ovulation in a dose‐dependent manner; it was reduced from 60.0% in the vehicle group to 38.5% in the 0.01 μM letrozole treated group, 6.67% in the 0.1 μM letrozole treated group, and 0.0% in the 1 μM letrozole treated group (*n* = 15,13,15,14, Cochran–Armitage trend test, *p* < 0.0001, Figure 4A). Next, to evaluate the effects of exogenous estrogen on the ovulation ability of letrozole‐treated follicles, we stimulated 0.1 μM letrozole‐treated follicles with 100 pg/mL or 250 pg/mL estradiol from Day 4 to Day 5 of the culture period. As shown in Figure 4B, exogenous estrogen improved the ovulation rate of 0.1 μM letrozole‐treated follicles from 4.2% to 16% at 100 pg/mL estradiol and 30% at 250 pg/mL estradiol (*n* = Cochran–Armitage test, *p* < 0.01, Figure 4B).

### 3.5 Effect of Letrozole on the Transcription of Ovulation‐Related Genes

**FIGURE 5:**
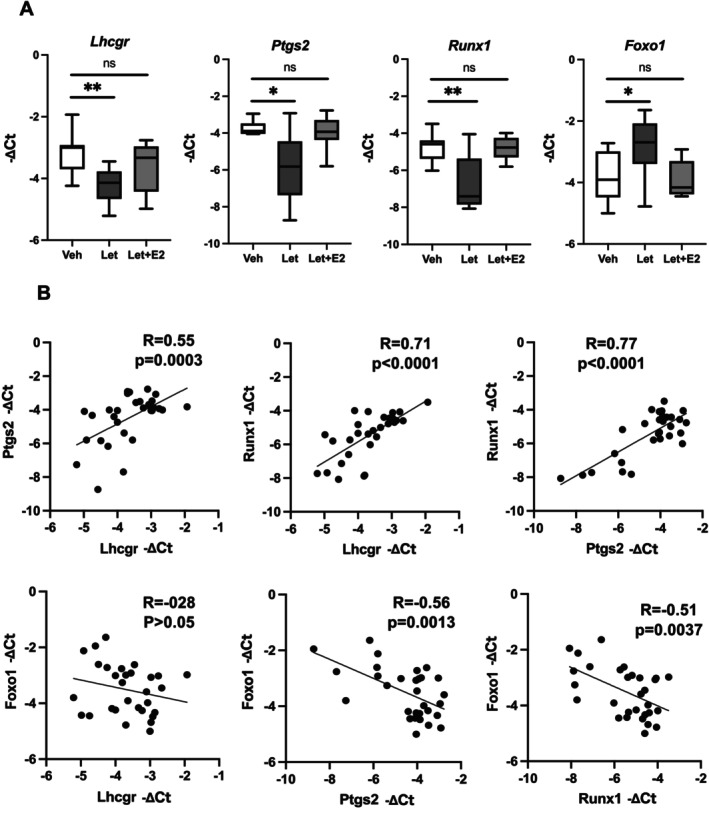
Effect of letrozole on the transcription of ovulation and luteinization‐related genes. The mRNA expression levels of three ovulation‐related genes (*Lhcgr*, *Ptgs2*, and *Runx1*) *and Foxo1* in 0.1 μM letrozole‐treated follicles were measured by RT‐qPCR 16 h after ovulation induction (hCG/hEGF stimulation). (A) Follicles treated with 0.1 μM letrozole showed significant reductions in the −Δ*C*_t_ values of *Lhcgr*, *Ptgs2*, and *Runx1* compared with vehicle‐treated follicles, whereas the −Δ*C*_t_ value of *Foxo1* was significantly increased. Co‐treatment with exogenous estradiol (E2) attenuated these changes. (B) Pearson's correlation analyses of −Δ*C*_t_ values demonstrated positive correlations between *Lhcgr* and *Ptgs2* (upper left panel; *R* = 0.55, *p* = 0.0003), between *Lhcgr* and *Runx1* (upper middle panel; *R* = 0.71, *p* < 0.0001), and between *Ptgs2* and *Runx1* (upper right panel; *R* = 0.77, *p* < 0.0001). In contrast, *Foxo1* − ΔCt values were negatively correlated with *Ptgs2* (lower middle panel; R = −0.56, *p* = 0.0013) and *Runx1* (lower right panel; R = −0.51, *p* = 0.0037), whereas the correlation with *Lhcgr* was not statistically significant (lower left panel; R = −0.28, *p* > 0.05). Lhcgr, luteinizing hormone/chorionic gonadotropin receptor; Ptgs2, prostaglandin‐endoperoxide synthase 2; Runx1, Runt‐related transcription factor 1; Foxo1, forkhead box protein O1.

To further examine how letrozole impairs in vitro ovulation, we measured the transcription of four ovulation/luteinization‐related genes (*Lhcgr, Ptgs2*, *Runx1, and Foxo1*) in the follicles by RT‐qPCR 16 h after ovulation induction. As presented in Figure 5A, 0.1 μM letrozole‐treated follicles showed significant reductions in the −Δ*C*_t_ values of *Lhcgr* (left panel, *p* < 0.01, Wilcoxon test), *Runx1* (center panel, *p* < 0.05, Wilcoxon test), and *Ptgs2* (right panel, *p* < 0.01, Wilcoxon test), indicating decreased expression compared with vehicle‐treated follicles. Exogenous estrogen addition restored the transcription of these genes to levels observed in the vehicle‐treated follicles. In contrast, *Foxo1* −∆*C*_t_ values were significantly increased in the letrozole‐treated group compared with the vehicle group (*p* < 0.05, Wilcoxon test), and this change was attenuated by E2 supplementation (Figure 5A). Furthermore, *Fshr* expression exhibited a similar pattern 16 h after hCG/hEGF stimulation: *Fshr* −∆*C*_t_ values were significantly increased in the letrozole‐treated group compared with the vehicle group, and co‐treatment with E2 tended to attenuate this change (Figure S2). We also observed a positive correlation between the −∆*C*_t_s of *Lhcgr* and *Ptgs2* (Figure 5B, upper left panel, *R* = 0.55, *p* < 0.001) as well as a strong positive correlation between the −∆*C*_t_s of *Lhcgr* and *Runx1* (Figure 5B, upper center panel, *R* = 0.71, *p* < 0.0001) and between the −∆*C*_t_s of *Ptgs2* and *Runx1* (Figure 5B, upper right panel, R = 0.77, *p* < 0.0001). In contrast, *Foxo1* −Δ*C*_t_ values showed a negative correlation with *Ptgs2* (Figure 5B, lower center panel, R = −0.56, *p* = 0.0013) and *Runx1* (Figure 5B, lower right panel, R = −0.51, *p* = 0.0037), whereas the correlation with *Lhcgr* was not statistically significant (Figure 5B, lower left panel, R = −0.28, *p* > 0.05).

## 4 Discussion

We investigated the effects of letrozole on follicle growth and ovulation ability using an in vitro culture system of mouse follicles. We clarified that (1) letrozole did not alter early antral follicle survival and growth; (2) letrozole suppressed in vitro ovulation in a dose‐dependent manner; (3) estrogen replacement restored ovulation disorders caused by letrozole; and (4) letrozole attenuated follicular *Lhcgr* transcription and its downstream signals, and estrogen replacement reversed the defect. These findings suggest that continuous letrozole exposure during follicular growth from the early antral to late antral stage alters subsequent peri‐ovulatory responsiveness in vitro, potentially through estrogen deficiency in cultured mouse follicles.

We used an individual in vitro culture system of mouse early antral follicles to assess the effect of letrozole on murine follicle growth and ovulation in vitro. The in vitro growth of early antral follicles is a useful and practical method to test various substances. In addition, *Cyp19a1* mRNA expression in the cultured follicles increased drastically during the culture period. Therefore, we considered that an in vitro culture system of follicles is suitable for investigating the effects of letrozole on follicle development and maturation. In this study, we used letrozole at 0.01–1 μM for mouse experiments. This experimental concentration range of letrozole is almost as low as the clinical one, although its peak concentration was 7 times higher than the highest concentration of the clinical range of letrozole (Cmax: approximately 0.15 μM, Femara Tablets 2.5 mg, interview form, Novartis Japan, Japan). Therefore, the concentration of letrozole used in our in vitro experiments was close to its clinical blood concentration and thus appropriate for evaluating follicle growth and ovulatory ability.

We found that letrozole did not affect follicle survival and growth in in vitro‐cultured mouse follicles. Although estrogen promotes follicle growth in the late follicular phase, the diameter did not change in letrozole‐treated follicles compared with that in the vehicle‐treated follicles. This discrepancy may be explained by increased *Fshr* transcription in follicles. Increased local follicle‐stimulating FSH responsiveness might result from the follicle‐stimulation action of aromatase inhibitors [6]. Aromatase inhibitors are expected to increase *Fshr* transcription due to the accumulation of androgens, which are substrates for estrogen synthesis [20]. We found that the *Fshr* mRNA level in 0.1 μM letrozole‐treated follicles was significantly higher than that in the vehicle group on day 4 (Figure S1), consistent with prior long‐term culture studies using a murine models [21]. In line with this, the proportion of morphologically normal follicles reaching ≥ 300 μm was numerically higher up to 0.1 μM letrozole, although the absolute difference was modest and no significant dose–response trend was detected. High *Fshr* expression may compensate for poor follicle growth caused by estrogen deficiency in letrozole‐treated follicles. Our study may provide a detailed understanding of the local effects of aromatase inhibitors as ovarian stimulation agents on follicle development.

We demonstrated that continuous letrozole treatment markedly reduced the ovulation rate, even when stimulated with hCG and hEGF. Furthermore, exogenous estrogen restored in vitro ovulation ability and improved *Lhcgr* mRNA expression and its downstream signaling pathways. Several mechanisms of *Lhcgr* transcription in mural granulosa cells have been reported, including those involving estrogen, transcriptional factors, cell signaling, miRNA, and epigenetic modifications [22, 23, 24, 25]. *Lhcgr* mRNA level was positively correlated with *Cyp19a1* mRNA levels and intrafollicular estradiol levels in human granulosa cells derived from small antral follicles [26]. We also confirmed that letrozole reduced *Ptgs2* and *Runx1* expression, which was restored by estrogen treatment. Transcription of both genes are rapidly induced in mural granulosa cells at the initiation of the ovulatory process in response to the LH surge [27]. Moreover, while *Ptgs2* KO mice have normal follicle growth, they are infertile due to impaired ovulation [28]. To further characterize peri‐ovulatory follicular responses, we next examined markers associated with luteinization. In peri‐ovulatory follicles, *Foxo1* and *Fshr* expression typically declines during luteinization in mice [29, 30]. Notably, mRNA levels of these genes remained elevated in the letrozole‐treated group 16 h after hCG/hEGF stimulation, and this tendency was attenuated by E2 supplementation. This pattern is consistent with an altered peri‐ovulatory/luteinization program under sustained aromatase inhibition. Because *Lhcgr* expression was assessed only 16 h after hCG/hEGF stimulation in the present study, our data do not directly determine whether letrozole impairs preovulatory acquisition of LH responsiveness before ovulatory stimulation. Therefore, we propose that the altered *Lhcgr* transcription and its downstream signaling cascades observed after ovulation induction may contribute to impaired ovulatory competence in letrozole‐treated follicles.

Further research is needed to determine whether our findings apply to humans. Our study provides important insights for the evaluation of oocyte cryopreservation in fertility preservation programs in women with breast cancer. Our findings reveal that continuous letrozole treatment reduced peri‐ovulatory responsiveness of follicles, under a protocol in which letrozole exposure was maintained throughout follicular growth in vitro (from the early antral stage toward the peri‐ovulatory stage), which differs from clinically stage‐restricted regimens in which letrozole is administered only during the early antral phase. This finding further implies that the letrozole combination COS protocol may have disadvantages in the subsequent reproductive processes, such as fertilization and preimplantation embryo development. The high developmental potential of oocytes is important for achieving a live baby, which is one of the most important outcomes of fertility preservation other than tumor recurrence. However, evaluating the developmental potential of the retrieved oocytes is challenging because fertility preservation of cancer survivors comprises a long period from oocyte cryopreservation to thawing, followed by insemination, embryo culture, and embryo transfer. Cobo et al. retrospectively analyzed the cumulative pregnancy rate of 80 patients who underwent embryo transfer as compared to that of elective oocyte cryopreservation patients [31]. The initial cohort of this study included approximately 72.8% of the patients who underwent oocyte retrieval by the letrozole‐based COS protocol. This study showed that the cumulative pregnancy rate was lower than the expected rate. There are few reports on the developmental potential of oocytes collected by continuous administration of letrozole in cancer patients [32, 33]. In other words, the interpretation of clinical outcomes may be confounded by differences in COS protocols. Additionally, gynecologists may need to educate patients about the limited evidence regarding the developmental potential of oocytes obtained from letrozole cycles, particularly when extrapolating across different letrozole exposure windows. Further research on ovulation stimulation using alternatives to letrozole‐based COS and the development of alternative methods for fertility preservation in ER‐positive cancer patients may also be necessary.

A limitation of this study is that androgen concentrations in the culture media were not directly measured. Previous studies have reported that excess or nonaromatizable androgens can attenuate FSH‐induced acquisition of LHCGR in granulosa cells and reduce follicular *Lhcgr* mRNA in vivo, suggesting that an androgenic milieu may negatively modulate LH/hCG responsiveness. In our model, pharmacological inhibition of androgen synthesis with abiraterone acetate failed to restore ovulation in letrozole‐treated follicles (Figure S3), whereas exogenous estrogen supplementation rescued both gene expression changes and the ovulatory phenotype. Together, these findings suggest that estrogen deficiency may be the primary determinant of LH receptor suppression in this in vitro model. Second, we did not systematically assess the nuclear maturation status of oocytes recovered after in vitro ovulation induction (GV/MI/MII). Because our assay was designed to evaluate follicle‐level ovulatory responsiveness and Lhcgr‐related signaling, future studies using an optimized culture workflow will be required to determine how letrozole and estrogen supplementation affect oocyte nuclear maturation in parallel with follicular outcomes.

In conclusion, our study reveals that clinical concentration of letrozole did not alter follicle growth, but dysregulated ovulation ability in in vitro‐grown mouse follicles. Letrozole reduced ovulatory responsiveness and was associated with decreased *Lhcgr* transcription 16 h after hCG stimulation, likely due to endogenous estrogen deficiency. Our study suggests that further investigation is warranted to determine whether the oocytes of cancer survivors retrieved using the letrozole combination COS protocols are acceptable for fertility preservation.

## Funding

The Japan Society for the Promotion of Science KAKENHI Grants 22 K16871 (to T.K.), 15 K10659 (to H.T.), and 25253092 (to M.S.) helped fund this study.

## Ethics Statement

All procedures involving animals were approved by the Institutional Animal Care and Use Committee of Chiba University (A4‐107). All institutional and national guidelines for the care and use of laboratory animals were followed.

## Conflicts of Interest

The authors declare no conflicts of interest.

## Supporting information


**Figure S1:** Fshr mRNA expression 0.1 μM letrozole‐treated follicle.

**Figure S2:** Fshr mRNA expression 16 h after ovulation induction in letrozoletreated follicles.

**Figure S3:** Effect of inhibition of de novo androgen synthesis on the ovulatory phenotype of letrozole‐treated follicles.

**Table S1:** qPCR Primer sequences.

## Data Availability

Research data are not shared.
