# Supplementary material for: Letrozole Reduces Ovulatory Responsiveness in In Vitro‐Grown Mouse Follicles
Source: Reprod Med Biol. 2026 Apr 15;25(1):e70047. doi: 10.1002/rmb2.70047 (PMC13081686; doi:10.1002/rmb2.70047)
Supplement: Supplementary file 1 — Figure S1: Fshr mRNA expression 0.1 μM letrozole‐treated follicle. [file RMB2-25-e70047-s002.pdf]

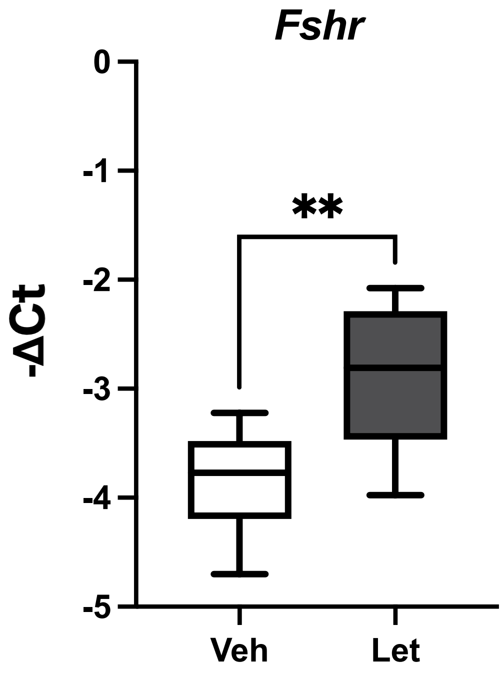

Supplemental Figure 1 *Fshr* mRNA expression 0.1 μM letrozole-treated follicles

RT-qPCR results of *Fshr* mRNA in 0.1 μM letrozole-treated follicles on day 4 of culture. We measured *Fshr* mRNA expression in vehicle groups and 0.1 μM letrozole-treated follicles on culture day 4. The -ΔCt of *Fshr* in 0.1 μM letrozole-treated follicles was significantly higher than that in vehicle-treated follicles (n=9, 11, **P<0.01, Wilcoxon-test). Fshr, follicle-stimulating hormone receptor; Let, letrozole; Veh, vehicle.
