# Supplementary material for: Letrozole Reduces Ovulatory Responsiveness in In Vitro‐Grown Mouse Follicles
Source: Reprod Med Biol. 2026 Apr 15;25(1):e70047. doi: 10.1002/rmb2.70047 (PMC13081686; doi:10.1002/rmb2.70047)
Supplement: Supplementary file 2 — Figure S2: Fshr mRNA expression 16 h after ovulation induction in letrozoletreated follicles. [file RMB2-25-e70047-s003.pdf]

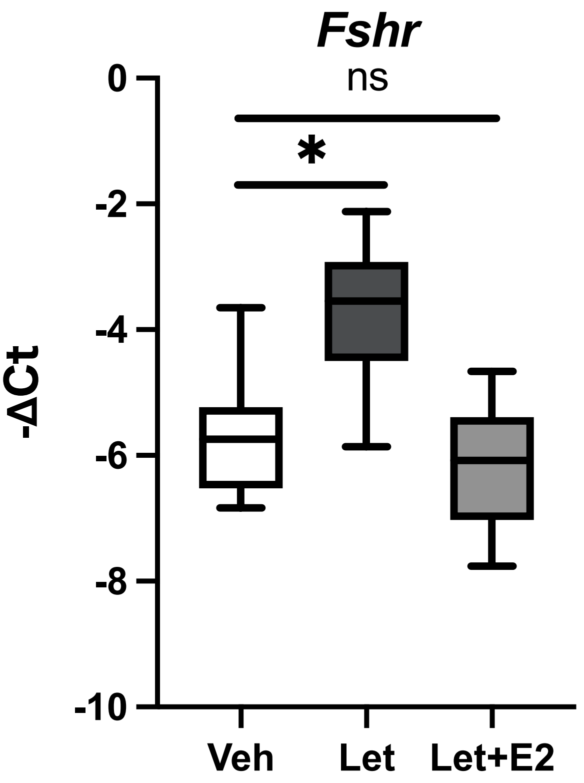

Supplemental Figure 2 *Fshr* mRNA expression 16 h after ovulation induction in letrozole-treated follicles.

The mRNA expression level of *Fshr* was measured by RT-qPCR in cultured mouse follicles 16 h after ovulation induction (hCG/EGF stimulation). Follicles were cultured under vehicle (Veh, n=11), 0.1 μM letrozole (Let, n=10), or 0.1 μM letrozole with estradiol supplementation (Let+E2, n=10). Data are presented as −ΔCt values normalized to *Actb*. Letrozole treatment significantly increased *Fshr* expression compared with the vehicle group, and this increase was attenuated by E2 co-treatment. $P < 0.05$; ns, not significant (Wilcoxon test).
Veh, vehicle; Let, letrozole; E2, estradiol; hCG, human chorionic gonadotropin; EGF, epidermal growth factor.
