# Supplementary material for: Letrozole Reduces Ovulatory Responsiveness in In Vitro‐Grown Mouse Follicles
Source: Reprod Med Biol. 2026 Apr 15;25(1):e70047. doi: 10.1002/rmb2.70047 (PMC13081686; doi:10.1002/rmb2.70047)
Supplement: Supplementary file 3 — Figure S3: Effect of inhibition of de novo androgen synthesis on the ovulatory phenotype of letrozole‐treated follicles. [file RMB2-25-e70047-s001.pdf]

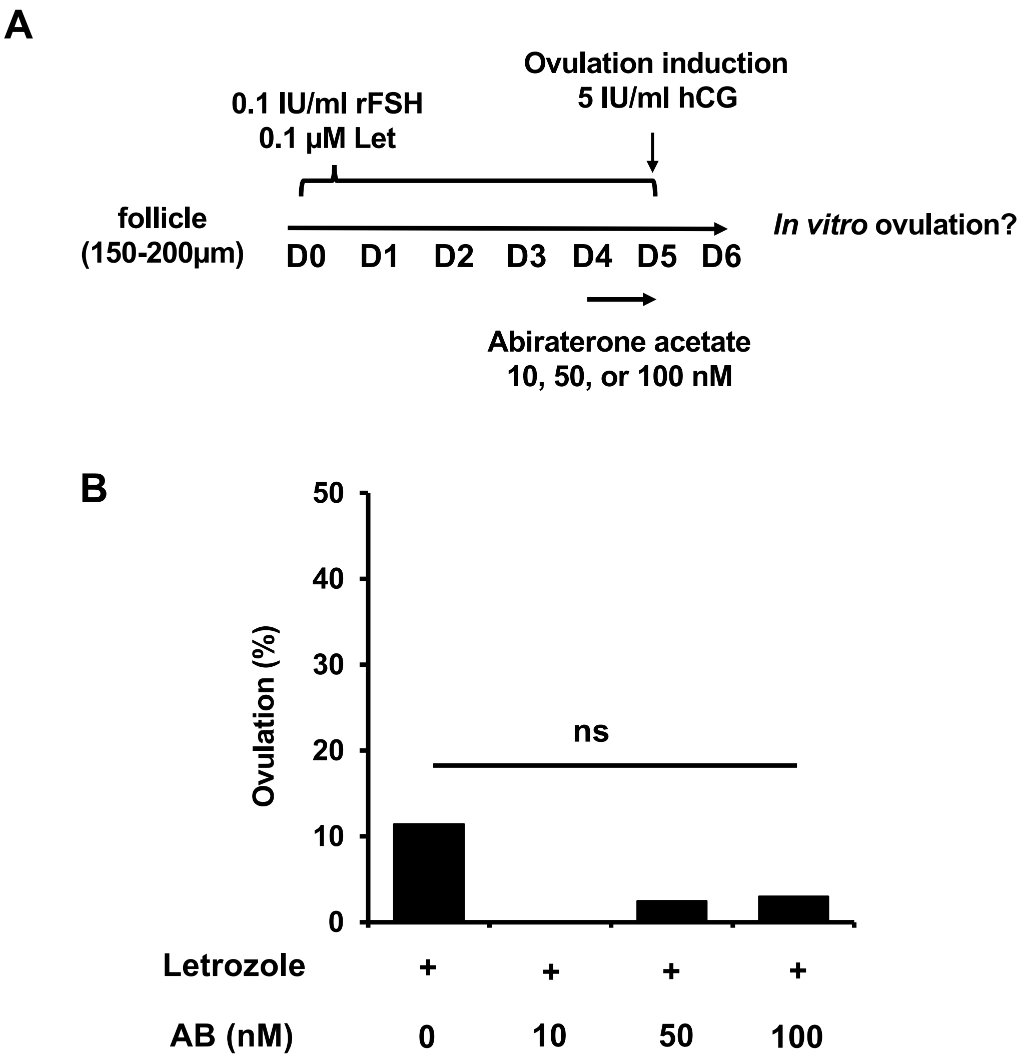

Supplemental Figure 3 Effect of inhibition of de novo androgen synthesis on the ovulatory phenotype of letrozole-treated follicles.

(A) Schematic overview of the ovulation-rescue experiment. Mouse early antral follicles (150–200 µm) were cultured with 0.1 IU/mL rFSH in the presence of 0.1 µM letrozole. Abiraterone acetate (AB; 10, 50, or 100 nM), a CYP17A1 inhibitor, was added from day 4 for 24 h (day 4 to day 5). Ovulation was induced on day 5 by addition of 5 IU/mL hCG and 5 ng/mL EGF, and assessed 16 h later.
(B) Co-treatment with AB did not significantly restore in vitro ovulation in letrozole-treated follicles. Data are shown as ovulation rate (%). Numbers of follicles analyzed were n = 35, 29, 40, and 33 for AB 0, 10, 50, and 100 nM, respectively; ns, not significant.
